# Epidemiology of glomerular diseases in Northeastern Brazil from 2008–2024

**DOI:** 10.1590/2175-8239-JBN-2025-0295en

**Published:** 2026-08-07

**Authors:** Andressa Monteiro Sodré, Marcos Adriano Garcia Campos, Érico Murilo Monteiro Cutrim, Pedro Manuel Barros de Sousa, Thina Klicia Mendonça Oliveira, Kwang Il Marciaga Teófilo, Felipe Leite Guedes, José Bruno de Almeida, Igor Denizarde Bacelar Marques, Cristianne da Silva Alexandre, Rafael Fernandes Vanderlei Vasco, Denizar Vianna Araújo, Francisco Rasiah Ladchumananandasivam, Giuseppe Cesare Gatto, Orlando Vieira Gomes, Denise Maria do Nascimento Costa, Douglas Rafanelle Moura de Santana, Monique Pereira Rêgo Muniz, Dyego José de Araújo Brito, Natalino Salgado, Precil Diego Miranda de Menezes Neves, Gyl Eanes Barros Silva

**Affiliations:** 1 Universidade Federal do Maranhão, Faculdade de Medicina, São Luís, MA, Brazil.; 2 Secretaria de Estado da Saúde do Maranhão, São Luís, MA, Brazil.; 3 Universidade Estadual Paulista, Faculdade de Medicina de Botucatu, Hospital das Clínicas, Botucatu, SP, Brazil.; 4 Universidade Federal do Maranhão, Hospital Universitário, São Luís, MA, Brazil.; 5 Universidade Federal do Rio Grande do Norte, Hospital Universitário, Natal, RN, Brazil.; 6 Universidade Federal do Piauí, Hospital Universitário, Serviço de Nefrologia, Teresina, PI, Brazil.; 7 Universidade Federal do Cariri, Clínica Escola, Juazeiro do Norte, CE, Brazil.; 8 Universidade Federal de Alagoas, Hospital Universitário, Maceió, AL, Brazil.; 9 Universidade do Estado do Rio de Janeiro, Rio de Janeiro, RJ, Brazil.; 10 Universidade Federal da Paraíba, Hospital Universitário, João Pessoa, PB, Brazil.; 11 Universidade de Brasília, Hospital Universitário, Brasília, DF, Brazil.; 12 Universidade Federal do Vale do São Francisco, Hospital Universitário, Petrolina, PE, Brazil.; 13 Universidade Federal de Pernambuco, Hospital das Clínicas, Recife, PE, Brazil.; 14 Universidade Federal de Sergipe, Hospital Universitário, Aracaju, SE, Brazil.; 15 Universidade de São Paulo, Hospital das Clínicas, São Paulo, SP, Brazil.

**Keywords:** Glomerulonephritis, Renal Insufficiency, Chronic, Epidemiology

## Abstract

**Introduction:**

Glomerulopathies are the third leading cause of dialysis-dependent chronic kidney disease (CKD) in Brazil and are most commonly diagnosed by kidney biopsy; however, data regarding the prevalence and epidemiological characteristics of glomerulopathies are scarce. The present article reports the results of an epidemiological survey of glomerular diseases in Northeast Brazil.

**Methods:**

This retrospective study, based on kidney biopsy data from all states in Northeast Brazil (2008–2024), was conducted at a reference center for kidney pathology. Demographic data (age, sex, and ethnicity), clinical history, indications for kidney biopsy, and histopathological diagnoses were collected. Frequencies are presented as percentages, along with maps and charts.

**Results:**

Data from 2,661 patients (median age, 31 years [interquartile range, 19–45 years]) were included in the study, with a slight female predominance (n = 1,533 [57.6%]). The indication for biopsy was identified in 85.9% of cases, with nephrotic syndrome being the most common (n = 1,075 [40.39%]). Lupus nephritis (LN) was the most frequent etiology (n = 876 [32.9%]), followed by focal segmental glomerulosclerosis (FSGS; n = 455 [17.1%]). The most common diagnosis among older adults was membranous nephropathy (n = 43 [18.8%]), whereas the most prevalent diagnosis among children was FSGS (n = 105 [24.5%]).

**Conclusion:**

The profile of glomerulopathies reflects not only local biopsy indications but also the heterogeneity of the population of Northeast Brazil and its particular ethnic and socioeconomic characteristics. Glomerulopathies, such as LN, accounted for the majority of cases, indicating the influence of ancestral factors in this region.

## INTRODUCTION

Glomerulopathies are among the leading causes of chronic kidney disease (CKD) worldwide and tend to progress to renal replacement therapy more rapidly than other forms of CKD^1,2,3^. According to the 2023 Census of the Brazilian Society of Nephrology, 5% of patients are diagnosed with glomerulonephritis at the time of dialysis initiation, which makes it the third most frequent underlying condition, after arterial hypertension and diabetes mellitus^3^. Moreover, its true prevalence is likely to be higher, considering the large number of CKD cases of unknown etiology. The scarcity of data in many regions and limited access to kidney biopsy contribute to significant gaps in the understanding of the epidemiological characteristics of glomerular diseases^4,5,6,7,8,9^.

This significant limitation in the number of kidney biopsies performed in many regions of Brazil, particularly in those with poor socioeconomic indicators, has hindered the development of population-based studies. In Brazil, few studies have investigated the clinical and epidemiological characteristics of glomerulopathies using large samples^7,10^, and long-term outcomes and associated risk factors remain difficult to assess in cohort studies^11^. Recently, the first report from the nationwide prospective registry of kidney biopsies in Brazil provided new insights into the clinical and pathological characteristics of glomerulopathies in nearly 1,000 patients^12^. However, the data were primarily derived from large centers and were updated through 2021.

Despite having some of the highest CKD rates, the Brazilian Northeast (composed of the states of Maranhão, Piauí, Ceará, Rio Grande do Norte, Paraíba, Pernambuco, Alagoas, Sergipe, and Bahia) has been the subject of very few populationbased studies, specifically those restricted to the states of Bahia^5^ and Pernambuco^8^. This region has a high prevalence of factors that significantly influence the epidemiological profile of glomerular diseases, such as the ancestry of ethnic groups with a higher prevalence of associated genetic mutations^13^, as well as unfavorable socioeconomic conditions and a high incidence of infectious diseases^10^.

The present investigation aimed to characterize the epidemiology of glomerular diseases in a large sample of kidney biopsies performed in Northeast Brazil.

## METHODS

A descriptive study investigating the characteristics of glomerular diseases in patients undergoing kidney biopsy at 10 tertiary referral centers in Northeast Brazil, distributed across the nine states of this region (namely, *Hospital Universitário da Universidade Federal do Maranhão* [São Luís, Maranhão], *Hospital Universitário Lauro Wanderley* [João Pessoa, Paraíba], *Hospital Universitário Alcides Carneiro* [Campina Grande, Paraíba], *Hospital Universitário Onofre Lopes* [Natal, Rio Grande do Norte], *Hospital Universitário Professor Alberto Antunes* [Maceió, Alagoas], *Hospital Universitário da Universidade Federal do Piauí* [Teresina, Piauí], *Hospital Universitário da Universidade Federal do Vale do São Francisco* [Petrolina, located on the Pernambuco–Bahia border], *Hospital Universitário da Universidade Federal de Sergipe* [Aracaju, Sergipe], *Hospital Regional do Cariri* [Cariri, Ceará], and *Hospital Universitário de Lagarto* [Lagarto, Sergipe]), was conducted between January 2008 and December 2024. Kidney biopsy samples were collected locally in the state of origin and sent for analysis and storage to the Immunofluorescence and Electron Microscopy Laboratory at the *Hospital Universitário da Universidade Federal do Maranhão* (São Luís, Brazil), a reference center for kidney biopsies serving the Federal University Hospitals within the Brazilian Hospital Network (*EBSERH* [Portuguese abbreviation]). All native kidney biopsies were analyzed, and transplant recipients and biopsies that did not contain renal cortex tissue were excluded.

Data used in this study were entered into the Electronic Biopsy Request System and into histopathological reports, including histopathological diagnosis, demographic data (age, sex, and ethnicity), and indications for kidney biopsy. Age was stratified as follows: ≤ 14 years (children and adolescents); 15–29 years (young adults); 30–59 years (adults); and ≥ 60 years (older adults). Indications for kidney biopsy were classified into six clinical syndromes: asymptomatic urinary abnormalities; nephrotic syndrome; nephritic syndrome; acute kidney injury; rapidly progressive glomerulonephritis; and CKD.

All kidney biopsies were analyzed using light microscopy (LM) and immunofluorescence (IF). However, only some cases underwent electron microscopy (EM), depending on the suspected etiology or when a diagnosis could not be established by LM/IF methods. For LM, kidney tissue was paraffin-embedded and stained with hematoxylin-eosin, Masson’s trichrome, periodic acid–Schiff (PAS), and methenamine silver. IF employed the direct technique on frozen tissue, using an antibody panel for immunoglobulins (IgA, IgG, IgM), complement fractions (C3, C1q), fibrinogen, and light chains. Inconclusive IF results were not considered in this analysis due to the low sample size or the absence of glomerular material (90 cases during the study period).

Ultrastructural analysis was performed using transmission EM after osmium fixation and resin embedding. As the EM procedure was not standardized across all participating centers, we chose not to describe it. When tissue was available, EM was performed in cases for which LM and IF were inconclusive.

Histological diagnoses were classified according to etiology, based on the predominant involvement identified in the biopsy and evaluated by the pathologist. In addition to conventional diagnoses of glomerular, tubulointerstitial, or vascular pathologies, the “Other Diagnoses” category primarily included genetic diseases of renal significance.

Data were tabulated and analyzed using R version 4.2.2 (R Core Team; R Foundation for Statistical Computing, Vienna, Austria). Data are presented in graphs and tables with absolute and relative frequencies. Missing data for each variable were disregarded when calculating frequencies. The significance level was set at 5% for all analyses. The chi-squared test was used to assess the association between the studied variables and sex and age.

The Research Ethics Committee of the *Hospital Universitário da Universidade Federal do Maranhão* approved this study (No. 4,750,825) and waived the requirement for informed consent.

## RESULTS

**Figure 1:**
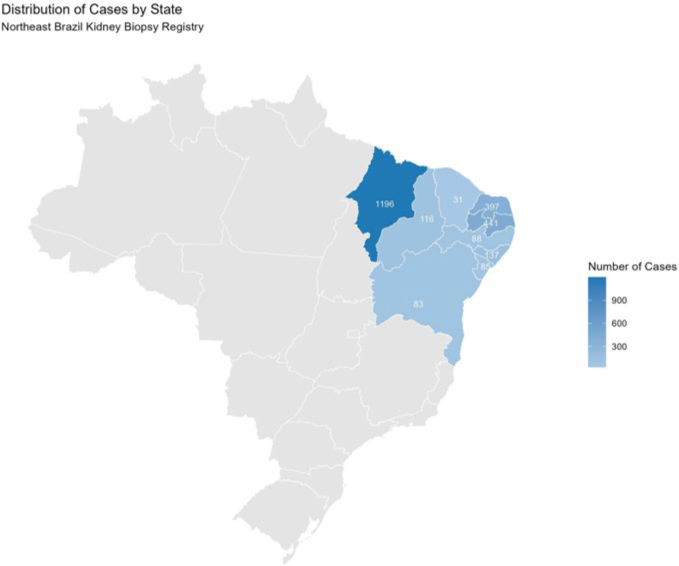
Distribution of the origin of kidney biopsy samples, Northeast Brazil (2008–2024).

A total of 2,911 patients underwent native kidney biopsy at the participating centers over a 16-year period (Figure 1). Patients with inconclusive biopsies and those without renal tissue samples were excluded (n = 250). Ultimately, data from 2,661 kidney biopsies were included. Female patients predominated (n = 1,533 [57.6%]); the median age was 31 years (interquartile range [IQR], 19–45 years). Demographically, the cohort was distributed as follows: children and adolescents, n = 428 (16.24%); young adults, n = 807 (30.62%); adults, n = 1,171 (44.44%); and older adults, n = 229 (8.69%). Ethnicity was identified in 2,261 (84.96%) patients, with a predominance of non-Caucasians (n = 1,706 [75.45%]). EM was performed on 146 (5%) kidney biopsy samples, which were used as a diagnostic aid for certain pathologies.

Clinical indications for biopsy were identified in 85.9% of cases, with nephrotic syndrome being the most common (n = 1,075 [40.39%]). Variations in biopsy indications according to sex and age are summarized in Table 1.

**Table 1:** Distribution of glomerular syndromes by sex and age.

Syndrome	Sex n (%)	p-value	Age group				p-value
			≤ 14 n (%)	15–29 n (%)	30–59 n (%)	≥ 60 n (%)		
Nephrotic Syndrome	M: 541 (50.3) F: 534 (49.7)	0.831	222 (20.7)	324 (30.3)	417 (39)	107 (10)	<0.001
Asymptomatic Urinary Abnormalities	M: 87 (29.6) F: 207 (70.4)	<0.001	36 (12.3)	83 (28.4)	158 (54.1)	15 (5.1)	<0.001
Nephritic/Nephrotic Syndrome	M: 105 (37.9) F: 172 (62.1)	<0.001	39 (14.1)	90 (32.5)	123 (44.4)	25 (9)	<0.001
Nephritic Syndrome	M: 60 (28) F: 154 (72)	<0.001	30 (14.1)	64 (30)	98 (46)	21 (9.9)	<0.001
Chronic Kidney Disease	M: 85 (44.3) F: 107 (55.7)	0.112	22 (11.6)	54 (28.4)	98 (51.6)	16 (8.4)	<0.001
Rapidly Progressive Glomerulopathy	M: 49 (34.5) F: 93 (65.5)	<0.001	15 (11.2)	50 (37.3)	60 (44.8)	9 (6.7)	<0.001
Acute Kidney Injury	M: 31 (37.8) F: 51 (62.2)	0.027	6 (7.4)	17 (21)	43 (53.1)	15 (18.5)	<0.001
Others	M: 6 (60) F: 4 (40)	0.527	2 (20)	2 (20)	5 (50)	1 (10)	0.308

Abbreviations – M: male; F: female.

**Figure 2:**
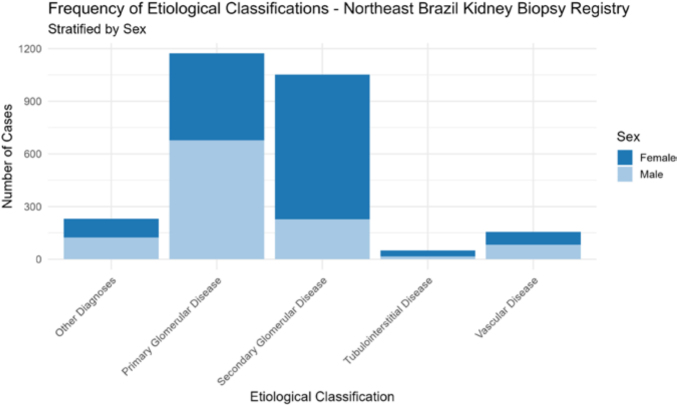
Classification of the etiology of kidney biopsy samples according to sex, Northeast Brazil (2008–2024).

Primary glomerular disease occurred in 1,153 (43.32%) cases, and secondary glomerular disease occurred in 1,052 (39.53%) (Figure 2). Among the study population, lupus nephritis (LN) was the predominant diagnosis (n = 876 [32.9%]) and was more common among adult women (n = 725 [82.8%]) and and non-White individuals (n = 567 [64.7%]). Focal segmental glomerulosclerosis (FSGS) was the second most frequent diagnosis (n = 455 [17.1%]), affecting individuals with a median age of 25 years (IQR, 14–41.5 years). Among older adults, the most common diagnosis was membranous nephropathy (n = 43 [18.8%]), whereas among children, the most prevalent diagnosis was FSGS (n = 105 [24.5%]) (Table 2).

**Table 2:** Distribution of histopathological diagnoses in a kidney biopsy registry from Northeastern Brazil.

Histopathological diagnosis	n	%	Median age (IQR)	Sex n/%	Ethnicity n/%
Lupus Nephritis	876	32.9	29 (21–39)	F: 725/82.8 M: 151/17.2	Non-White: 567/64.7 White: 177/20.2
Focal Segmental Glomerulosclerosis	455	17.1	25 (14–41.5)	F: 195/42.9 M: 260/57.1	Non-White: 305/67 White: 91/20
Membranous Nephropathy	252	9.5	44 (31–55)	F: 102/40.5 M: 150/59.5	Non-White: 167/66.3 White: 50/19.8
IgA Nephropathy	115	4.3	35 (26.5–44)	F: 51/44.3 M: 64/55.7	Non-White: 63/54.8 White: 35/30.4
Collapsing Glomerulopathy	106	4.0	25 (16–37)	F: 49/46.2 M: 57/53.8	Non-White: 66/62.3 White: 13/12.3
Minimal Change Disease	91	3.4	15 (8–27.75)	F: 35/38.5 M: 56/61.5	Non-White: 57/62.6 White: 25/27.5
Diabetic Nephropathy	90	3.4	54 (45–62)	F: 39/43.3 M: 51/56.7	Non-White: 60/66.7 White: 17/18.9
Minimal Change Disease/Focal Segmental Glomerulosclerosis	67	2.5	21 (9.5–37)	F: 28/41.8 M: 39/58.2	Non-White: 37/55.2 White: 25/37.3
Acute Diffuse Glomerulonephritis	56	2.1	26 (15–44)	F: 25/44.6 M: 31/55.4	Non-White: 36/64.3 White: 12/21.4
Systemic Vasculitis	55	2.1	43 (24–56)	F: 32/58.2 M: 23/41.8	Non-White: 34/61.8 White: 10/18.2
Acute Tubular Necrosis	50	1.9	47 (22–60)	F: 33/66 M: 17/34	Non-White: 30/60 White: 9/18
Crescentic Glomerulonephritis	35	1.3	42 (23.5–50)	F: 19/54.3 M: 16/45.7	Non-White: 26/83.9 White: 5/16.1
Monoclonal Gammopathy	35	1.3	56 (45–63.5)	F: 21/60 M: 14/40	Non-White: 20/57.1 White: 10/28.6
Thrombotic Microangiopathy	34	1.3	30 (16.75–36.75)	F: 20/58.8 M: 14/41.2	Non-White: 28/82.4 White: 5/14.7
Chronic Sclerosing Glomerulonephritis	25	0.9	41 (24–52	F: 12/48 M: 13/52	Non-White: 19/76 White: 6/24
Membranoproliferative Glomerulonephritis	24	0.9	44.5 (20.25–58.5)	F: 10/41.7 M: 14/58.3	Non-White: 14/58.3 White: 9/37.5
Benign Nephrosclerosis	20	0.8	44 (34.75–57)	F: 8/40 M: 12/60	Non-White: 13/65 White: 4/20
Chronic Nephropathy	18	0.7	33.5 (16.25–44)	F: 11/61.1 M: 7/38.9	Non-White: 14/77.8 White: 4/22.2
Atherosclerosis	10	0.4	32.5 (21–38.75)	F: 5/50 M: 5/50	Non-White: 6/60 White: 4/40
Diffuse Mesangial Sclerosis	7	0.3	5 (1.5–11)	F: 1/14.3 M: 6/85.7	Non-White: 4/57.1 White: 3/42.9
Others	11	0.4	39 (24.25–47.75)	F: 7/63.6 M: 4/36.4	Non-White: 4/36.4 White: 3/27.3

Abbreviations – IQR: interquartile range; F: female; M: male.

**Figure 3:**
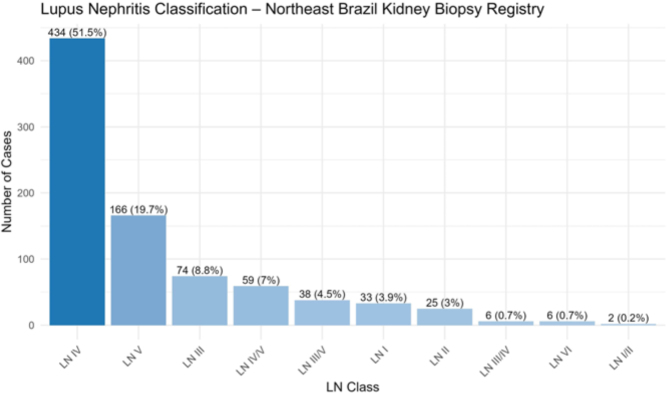
Frequency of lupus nephritis classes in kidney biopsy samples, Northeast Brazil (2008–2024).

The distribution according to LN class exhibited a higher frequency of class IV (51.5%), followed by class V (19.7%) (Figure 3).

Table 3 describes the main characteristics of the two most prevalent diagnoses in the study: FSGS and lupus nephritis. While FSGS showed a similar distribution across almost all age groups and among both sexes, lupus nephritis accounted for 89% of cases in the 15–59-year age group, with 83% being women. FSGS most commonly presented as nephrotic syndrome (72%), whereas lupus nephritis showed diverse clinical manifestations, including acute cases such as rapidly progressive glomerulonephritis in 6.2% of patients.

**Table 3:** Demographic and clinical characteristics of the two most prevalent diagnoses in a kidney biopsy registry from the Northeast region of Brazil (2008–2024): focal segmental glomerulosclerosis and lupus nephritis.

Characteristics	Focal segmental glomerulosclerosis (n = 455)	Lupus nephritis (n = 876)
**Age Group (years)**
≤ 14	124 (27%)	74 (8.5%)
15–29	131 (29%)	367 (42%)
30–59	164 (36%)	413 (47%)
≥ 60	32 (7.1%)	16 (1.8%)
**Sex**
Female	195 (43%)	725 (83%)
Male	260 (57%)	151 (17%)
**Ethnicity**
Non-White	305 (77%)	567 (76%)
White	91 (23%)	177 (24%)
**Syndrome**
Acute Kidney Injury	4 (1.0%)	19 (2.5%)
Chronic Kidney Disease	36 (9.2%)	54 (7.1%)
Asymptomatic Urinary Abnormalities	29 (7.4%)	142 (19%)
Nephritic Syndrome	10 (2.5%)	121 (16%)
Nephrotic Syndrome	283 (72%)	219 (29%)
Nephrotic/Nephritic Syndrome	24 (6.1%)	153 (20%)
Rapidly Progressive Glomerulonephritis	6 (1.5%)	47 (6.2%)
Others	1 (0.3%)	4 (0.5%)

## DISCUSSION

Only a few studies have addressed the epidemiological characteristics of patients with glomerular diseases in the Northeast region of Brazil^5,8^. The specific sociodemographic characteristics of this region, in turn, make it important to develop kidney biopsy registries in this area as a benchmark for comparison with other regions of the country and even with populations in other parts of the world.

Due to the high cost and significant social impact of kidney diseases, countries such as Japan^14^ and Italy^15^ regularly maintain national registries of glomerular diseases, a practice that is difficult to implement in Brazil because it is a continental country with substantial sociodemographic variability across regions^16,17^. Using a large population-based approach, we characterized patients who underwent kidney biopsy at public hospitals in Northeast Brazil. Of the 2,911 patients initially selected, a diagnosis could not be established in 8.58%, a rate lower than those reported in other studies, which ranged from 12% to 16%^4,5,10^.

The sex distribution was similiar to that observed in the 2010 Brazilian Registry of Kidney Biopsies^7^. The affected patients were generally adults and young adults, similar to the demographic profile reported in other studies^5,7,9,10^, which could be explained by the prevalence of the main glomerular diseases in this age group, such as LN and FSGS^18,19^.

Collapsing glomerulopathy (CG) disproportionately affects individuals of African descent, a phenomenon largely attributed to the presence of *APOL1* risk variants, which significantly increase susceptibility to developing this condition^20^. In addition to the genetic component, CG can be triggered by various environmental and clinical factors, including viral infections (HIV, parvovirus B19, and arboviruses), the use of certain medications, autoimmune diseases, neoplasms, ischemic events (such as thrombotic microangiopathy, peri-infarction lesions, and cholesterol embolism), and factors associated with kidney transplantation^21^. In the present study, 106 (4%) patients were diagnosed with CG. These findings reinforce the relevance of CG in the Brazilian setting and provide an estimate of the prevalence of this disease in the Northeast region of Brazil^22^.

Nephrotic syndrome was predominant among the elderly, with membranous nephropathy being the most common diagnosis in this group. Studies published in Brazil^23^ and the United Kingdom^24^ reported similar findings, contrasting with data published in the United States^25^ and Spain^26^, where acute kidney injury was the most common condition, and the most prevalent diagnosis was crescentic and pauci-immune glomerulopathy. However, the main indications for kidney biopsy in this age group are hematuria, asymptomatic proteinuria, nephrotic syndrome, rapidly progressive glomerulonephritis, acute kidney injury, or idiopathic CKD^24,27^. The association between membranous nephropathy and malignancy in the elderly population is well known^28^.

In Northeast Brazil^5,7,8,9,10^ and worldwide^29,30,31^, the primary indication for kidney biopsy is nephrotic syndrome^32^. The literature on nephrotic syndrome indicates that 60% to 70% of cases have a primary etiology, with FSGS and membranous nephropathy being the most common diagnoses among adult and older adult patients^33^, whereas minimal change disease and FSGS predominate among children and adolescents, representing 80% of cases^34^. LN was the most prevalent pathology among all other indications for kidney biopsy.

Primary glomerulopathy was more common than secondary glomerulopathy, with FSGS being the most prevalent among cases of primary glomerulopathy and LN among cases of secondary glomerulopathy. These findings are similar to those reported in several national studies^4,5,7,8^ and in studies from Latin America^35,36,37^. FSGS is the most common finding in the Americas, including the United States^18^ and Brazil^7,10^.

In Asia and Europe, immunoglobulin (Ig) A nephropathy is the most common primary glomerulopathy^6,37^. Although IgA nephropathy has shown a greater increase in prevalence over the past 15 years in Brazil^7^, it was identified in < 5% of the sample. This prevalence is lower than both the national estimate^7^ and the prevalence reported in another study conducted in the state of São Paulo (Brazil)^4^, which was approximately 10%. Compared with international studies, the reported incidence is even higher, ranging from 20% to 45%^29,35,36,38^. Factors related to colonization and ethnicity in the Northeast region, where there is a higher proportion of individuals of African descent—a population in which the prevalence of IgA nephropathy is lower^39^—, in addition to the lack of screening associated with delayed referral to a nephrologist and the criteria for biopsy indication applied in hospital services, may explain the reported results^37^. The high prevalence of LN may be related to the ethnic characteristics of the sample, with the majority of patients being of non-White ethnicity^40^.

The most prevalent histological types of LN were classes IV and V, a common finding reported in a review published in 2018^41^. The predominance of these classes could be explained by the selection criteria, according to which kidney biopsy is generally performed in patients with more severe kidney involvement, which is typical of these classes.

A low incidence of post-infectious glomerulonephritis was observed, even though it is an important cause of glomerulopathy in underdeveloped countries, given that the Northeast is the poorest region in the country, where sanitary conditions may be precarious. This could be explained by its often benign course, with renal manifestations occurring late in the infectious process and often without an indication for kidney biopsy^42^.

The low prevalence of genetic diseases, such as Alport syndrome, can be explained by the lack of standardization of specific methods, such as molecular tests and EM^43,44^.

Primary arterial hypertension is not an indication for kidney biopsy, and the finding of hypertensive nephrosclerosis on kidney biopsy results is uncommon, occurring in 1%–3.5% of patients^7,14,36^, which is similar to the results of our study. Arterial hypertension is the second leading cause of CKD in Europe and the main cause in Brazil^45^.

Crescentic glomerulonephritis occurs mainly in secondary glomerulopathy (90%), with LN and pauci-immune disease being the most common etiologies, as reported in the literature^46,47^.

Limitations of the present study include the lack of integration among the systems used by participating centers, which made it difficult to access patient prognostic information from a single electronic medical record, as well as the absence of a standardized protocol for performing EM on most kidney biopsy samples. The absence of some demographic data was also noted, highlighting the need for integrated records between the centers.

This was the first multicenter study of its kind in the Northeastern region of Brazil to describe the clinical and epidemiological characteristics of patients with glomerulopathies. Our results lay the groundwork for the development of a future glomerulopathy registry for the region. The future development of a computational tool to optimize kidney biopsy requests could help standardize the information relevant to populationbased studies.

## CONCLUSION

The presence of glomerulopathy was similar between males and females, with nephrotic syndrome being the most common indication for kidney biopsy. In contrast to most studies conducted in Brazil, LN was the most prevalent glomerulopathy, followed by FSGS. The findings of this study differ from records in southeastern and southern Brazil regarding the low prevalence of IgA nephropathy.. The profile of glomerulopathies reflects the indications for kidney biopsy and the heterogeneity of the Brazilian population, with diverse ethnic and socioeconomic characteristics. The results of this study may contribute to a better understanding of the epidemiology of glomerulopathies in Brazil.

## Data Availability

The datasets generated and/or analyzed during the current study are available from the corresponding author upon reasonable request.
