# Supplementary material for: Epidemiology of glomerular diseases in Northeastern Brazil from 2008–2024
Source: J Bras Nefrol. 2026 Aug 7;48(4):e20250295. doi: 10.1590/2175-8239-JBN-2025-0295en (PMC13456060; doi:10.1590/2175-8239-JBN-2025-0295en)
Supplement: Translation

# Translation: Epidemiologia das doenças glomerulares no Nordeste do Brasil de 2008 a 2024

**DOI:** 10.1590/2175-8239-JBN-2025-0295pt

**Authors:** Andressa Monteiro Sodré, Marcos Adriano Garcia Campos, Érico Murilo Monteiro Cutrim, Pedro Manuel Barros de Sousa, Thina Klicia Mendonça Oliveira, Kwang Il Marciaga Teófilo, Felipe Leite Guedes, José Bruno de Almeida, Igor Denizarde Bacelar Marques, Cristianne da Silva Alexandre, Rafael Fernandes Vanderlei Vasco, Denizar Vianna Araújo, Francisco Rasiah Ladchumananandasivam, Giuseppe Cesare Gatto, Orlando Vieira Gomes, Denise Maria do Nascimento Costa, Douglas Rafanelle Moura de Santana, Monique Pereira Rêgo Muniz, Dyego José de Araújo Brito, Natalino Salgado, Precil Diego Miranda de Menezes Neves, Gyl Eanes Barros Silva

**Affiliations:** 1 Universidade Federal do Maranhão, Faculdade de Medicina, São Luís, MA, Brasil.; 2 Secretaria de Estado da Saúde do Maranhão, São Luís, MA, Brasil.; 3 Universidade Estadual Paulista, Faculdade de Medicina de Botucatu, Hospital das Clínicas, Botucatu, SP, Brasil.; 4 Universidade Federa do Maranhão, Hospital Universitário, São Luís, MA, Brasil.; 5 Universidade Federa do Rio Grande do Norte, Hospital Universitário, Natal, RN, Brasil.; 6 Universidade Federa do Piauí, Hospital Universitário, Serviço de Nefrologia, Teresina, PI, Brasil.; 7 Universidade Federa do Cariri, Clínica Escola, Juazeiro do Norte, CE, Brasil.; 8 Universidade Federa de Alagoas, Hospital Universitário, Maceió, AL, Brasil.; 9 Universidade do Estado do Rio de Janeiro, Rio de Janeiro, RJ, Brasil.; 10 Universidade Federal da Paraíba, Hospital Universitário, João Pessoa, PB, Brasil.; 11 Universidade de Brasília, Hospital Universitário, Brasília, DF, Brasil.; 12 Universidade Federal do Vale do São Francisco, Hospital Universitário, Petrolina, PE, Brasil.; 13 Universidade Federal de Pernambuco, Hospital das Clínicas, Recife, PE, Brasil.; 14 Universidade Federal de Sergipe, Hospital Universitário, Aracaju, SE, Brasil.; 15 Universidade de São Paulo, Hospital das Clínicas, São Paulo, SP, Brasil.

**Keywords:** Glomerulonefrite, Insuficiência Renal Crônica, Epidemiologia

## Abstract

**Introdução:**

As glomerulopatias são a terceira principal causa de doença renal crônica (DRC) dependente de diálise no Brasil e são diagnosticadas mais comumente por meio de biópsia renal; no entanto, os dados referentes à prevalência e às características epidemiológicas das glomerulopatias são escassos. O presente artigo relata os resultados de um levantamento epidemiológico de doenças glomerulares no Nordeste do Brasil.

**Métodos:**

Este estudo retrospectivo, baseado em dados de biópsias renais de todos os estados do Nordeste do Brasil (2008–2024), foi conduzido em um centro de referência em patologia renal. Foram coletados dados demográficos (idade, sexo e etnia), histórico clínico, indicações para biópsia renal e diagnósticos histopatológicos. As frequências são apresentadas em porcentagens, com a geração de mapas e gráficos.

**Resultados:**

Foram incluídos no estudo dados de 2.661 pacientes (idade mediana de 31 anos; intervalo interquartil [IIQ],19–45), com leve predominância do sexo feminino (n = 1.533 [57,60%]). A indicação para biópsia foi identificada em 85,9% dos casos, sendo a síndrome nefrótica a mais comum (n = 1.075 [40,39%]). A nefrite lúpica (NL) foi a etiologia mais frequente (n = 876 [32,9%]), seguida pela glomeruloesclerose segmentar focal (GESF; n = 455 [17,1%]). O diagnóstico mais comum entre os idosos foi nefropatia membranosa (n = 43 [18,8%]), enquanto o diagnóstico mais prevalente entre as crianças foi GESF (n = 105 [24,5%]).

**Conclusão:**

O perfil de glomerulopatias reflete não apenas as indicações locais para biópsia, mas também a heterogeneidade da população do Nordeste do Brasil e suas características étnicas e socioeconômicas particulares. Glomerulopatias, como a NL, representaram a maioria dos casos, indicando a influência de fatores ancestrais nessa região.

## INTRODUÇÃO

As glomerulopatias estão entre as principais causas de doença renal crônica (DRC) em todo o mundo e tendem a progredir para terapia renal substitutiva mais precocemente do que outras formas de DRC^1,2,3^. De acordo com o Censo de 2023 da Sociedade Brasileira de Nefrologia, 5% dos pacientes recebem o diagnóstico de glomerulonefrite no momento do início da diálise, o que a torna a terceira condição subjacente mais frequente, depois da hipertensão arterial e do diabetes mellitus^3^. Além disso, sua prevalência real provavelmente é maior, considerando o grande número de casos de DRC de etiologia desconhecida. A escassez de dados em muitas regiões e o acesso limitado à biópsia renal contribuem para lacunas significativas na compreensão das características epidemiológicas das doenças glomerulares^4,5,6,7,8,9^.

Essa limitação significativa no número de biópsias renais realizadas em muitas regiões do Brasil, tipicamente naquelas com indicadores socioeconômicos desfavoráveis, tem dificultado o desenvolvimento de estudos populacionais. No Brasil, poucos estudos investigaram as características clínicas e epidemiológicas das glomerulopatias utilizando grandes amostras^7,10^, e os desfechos de longo prazo e os fatores de risco associados permanecem difíceis de avaliar em estudos de coorte^11^. Recentemente, o primeiro registro nacional prospectivo de biópsias renais no Brasil forneceu novas perspectivas sobre as características clínicas e patológicas das glomerulopatias em quase 1.000 pacientes^12^. No entanto, os dados foram provenientes principalmente de grandes centros e atualizados até 2021.

Apesar de apresentar algumas das maiores taxas de DRC, o Nordeste brasileiro (composto pelos estados do Maranhão, Piauí, Ceará, Rio Grande do Norte, Paraíba, Pernambuco, Alagoas, Sergipe e Bahia) tem sido objeto de poucos estudos populacionais, especificamente pesquisas restritas aos estados da Bahia^5^ e de Pernambuco^8^. Essa região apresenta alta prevalência de fatores que influenciam significativamente o perfil epidemiológico das doenças glomerulares, como a ancestralidade de grupos étnicos com maior prevalência de mutações genéticas associadas^13^, além de condições socioeconômicas desfavoráveis e alta incidência de doenças infecciosas^10^.

A presente investigação teve como objetivo caracterizar a epidemiologia das doenças glomerulares em uma grande amostra de biópsias renais realizadas na região Nordeste do Brasil.

## MÉTODOS

Estudo descritivo que investigou as características das doenças glomerulares em pacientes submetidos à biópsia renal em 10 centros de referência terciários da região Nordeste do Brasil, distribuídos pelos nove estados dessa região (a saber, Hospital Universitário da Universidade Federal do Maranhão [São Luís, Maranhão], Hospital Universitário Lauro Wanderley [João Pessoa, Paraíba], Hospital Universitário Alcides Carneiro [Campina Grande, Paraíba], Hospital Universitário Onofre Lopes [Natal, Rio Grande do Norte], Hospital Universitário Professor Alberto Antunes [Maceió, Alagoas], Hospital Universitário da Universidade Federal do Piauí [Teresina, Piauí], Hospital Universitário da Universidade Federal do Vale do São Francisco [Petrolina, divisa entre Pernambuco e Bahia], Hospital Universitário da Universidade Federal de Sergipe [Aracaju, Sergipe], Hospital Regional do Cariri [Cariri, Ceará] e Hospital Universitário de Lagarto [Lagarto, Sergipe]) entre janeiro de 2008 e dezembro de 2024. Amostras de biópsia renal foram coletadas localmente no estado de origem e enviadas para análise e armazenamento ao Laboratório de Imunofluorescência e Microscopia Eletrônica do Hospital Universitário da Universidade Federal do Maranhão (São Luís, Brasil), um centro de referência para biópsias renais dos Hospitais Universitários Federais da Rede Hospitalar Brasileira (EBSERH). Todas as biópsias renais nativas foram analisadas, e pacientes transplantados e biópsias que não atingiram o córtex renal foram excluídos.

Os dados utilizados neste estudo foram inseridos no Sistema Eletrônico de Solicitação de Biópsia e em laudos histopatológicos, incluindo diagnóstico histopatológico, dados demográficos (idade, sexo e etnia) e indicações para biópsia renal. A idade foi estratificada da seguinte forma: ≤ 14 anos (crianças e adolescentes); 15–29 anos (adultos jovens); 30–59 anos (adultos); e ≥ 60 anos (idosos). As indicações para biópsia renal foram classificadas em seis síndromes clínicas: anormalidades urinárias assintomáticas; síndrome nefrótica; síndrome nefrítica; lesão renal aguda; glomerulonefrite rapidamente progressiva; e DRC.

Todas as biópsias renais foram submetidas à microscopia óptica (MO) e à análise de imunofluorescência (IF). No entanto, apenas alguns casos foram submetidos à microscopia eletrônica (ME), dependendo da etiologia suspeita ou da impossibilidade de estabelecimento do diagnóstico pelos métodos de MO/IF. Para a MO, o tecido renal foi processado em parafina e corado com hematoxilina-eosina, tricrômico de Masson, PAS e prata metenamina. A IF empregou a técnica direta em tecido congelado, utilizando um painel de anticorpos para imunoglobulinas (IgA, IgG, IgM), frações do complemento (C3, C1q), fibrinogênio e cadeias leves. Resultados inconclusivos de imunofluorescência não foram considerados nesta análise devido ao tamanho reduzido da amostra ou à ausência de material glomerular (90 casos durante o período do estudo).

A análise ultraestrutural foi realizada por meio de ME de transmissão após fixação em ósmio e inclusão em resina. Como o procedimento de ME não foi padronizado em todos os centros participantes, optamos por não descrevê-lo.

Os diagnósticos histológicos foram divididos em cinco grupos: glomerulopatia primária; glomerulopatia secundária; doença tubulointersticial; doença vascular; e outros diagnósticos.

Os dados foram tabulados e analisados utilizando o software R versão 4.2.2 (*R Core Team*; *R Foundation for Statistical Computing*, Viena, Áustria). A apresentação dos dados foi feita por meio de gráficos e tabelas com frequências absolutas e relativas. Os dados faltantes para cada variável foram desconsiderados no cálculo das frequências. Para todas as análises, o nível de significância foi definido em 5%. O teste do qui-quadrado foi utilizado para avaliar a associação entre as variáveis estudadas e o sexo e a idade.

Este estudo foi aprovado pelo Comitê de Ética em Pesquisa do Hospital Universitário da Universidade Federal do Maranhão (Nº 4.750.825), que dispensou a exigência de consentimento livre e esclarecido.

## RESULTADOS

Um total de 2.911 pacientes foi submetido à biópsia renal nos centros participantes ao longo de um período de 16 anos (Figura 1). Pacientes com biópsias inconclusivas e aqueles sem amostras de tecido renal foram excluídos (n = 250). Ao final, foram incluídos dados de 2.661 biópsias renais. O sexo feminino foi predominante (n = 1.533 [57,6%]); a mediana de idade foi de 31 anos (intervalo interquartil [IIQ], 19–45 anos). Demograficamente, a coorte foi distribuída da seguinte forma: crianças e adolescentes, n = 428 (16,24%); adultos jovens, n = 807 (30,62%); adultos, n = 1.171 (44,44%); e idosos, n = 229 (8,69%). A etnia foi identificada em 2.261 (84,96%) pacientes, com predominância de não caucasianos (n = 1.706 [75,45%]). A ME foi realizada em 146 (5%) biópsias renais, utilizadas como auxílio diagnóstico em algumas patologias.

**Figura 1:** Distribuição da origem das amostras de biópsia renal, Nordeste do Brasil (2008–2024).

As indicações clínicas para biópsia foram identificadas em 85,9% dos casos, sendo a síndrome nefrótica a mais comum (n = 1.075 [40,39%]). As variações nas indicações de biópsia de acordo com sexo e idade estão resumidas na Tabela 1.

**Tabela 1:** Distribuição da síndrome glomerular por sexo e faixa etária.

Síndrome	Sexo n (%)	Valor de p	Faixa etária				Valor de p
			≤ 14 n (%)	15–29 n (%)	30–59 n (%)	≥ 60 n (%)	
Síndrome Nefrótica	M: 541 (50,3) F: 534 (49,7)	0,831	222 (20,7)	324 (30,3)	417 (39)	107 (10)	<0,001
Anormalidades Urinárias Assintomáticas	M: 87 (29,6) F: 207 (70,4)	<0,001	36 (12,3)	83 (28,4)	158 (54,1)	15 (5,1)	<0,001
Síndrome Nefrítica/Nefrótica	M: 105 (37,9) F: 172 (62,1)	<0,001	39 (14,1)	90 (32,5)	123 (44,4)	25 (9)	<0,001
Síndrome Nefrítica	M: 60 (28) F: 154 (72)	<0,001	30 (14,1)	64 (30)	98 (46)	21 (9,9)	<0,001
Doença Renal Crônica	M: 85 (44,3) F: 107 (55,7)	0,112	22 (11,6)	54 (28,4)	98 (51,6)	16 (8,4)	<0,001
Glomerulopatia Rapidamente Progressiva	M: 49 (34,5) F: 93 (65,5)	<0,001	15 (11,2)	50 (37,3)	60 (44,8)	9 (6,7)	<0,001
Lesão Renal Aguda	M: 31 (37,8) F: 51 (62,2)	0,027	6 (7,4)	17 (21)	43 (53,1)	15 (18,5)	<0,001
Outros	M: 6 (60) F: 4 (40)	0,527	2 (20)	2 (20)	5 (50)	1 (10)	0,308

Abreviaturas – M: masculino; F: feminino.

A doença glomerular primária ocorreu em 1.153 (43,32%) casos, e a doença glomerular secundária ocorreu em 1.052 (39,53%) (Figura 2). Entre a população do estudo, a nefrite lúpica (NL) foi o diagnóstico predominante (n = 876 [32,9%]) e foi mais comum entre mulheres adultas (n = 725 [82,8%]) e pacientes de etnia não branca (n = 567 [64,7%]). A glomeruloesclerose segmentar focal (GESF) foi o segundo diagnóstico mais frequente (n = 455 [17,1%]), afetando indivíduos com idade mediana de 25 anos (IIQ, 14–41,5). Entre os idosos, o diagnóstico mais comum foi nefropatia membranosa (n = 43 [18,8%]), enquanto, entre as crianças, o diagnóstico mais prevalente foi GESF (n = 105 [24,5%]) (Tabela 2).

**Figura 2:** Classificação da etiologia das amostras de biópsia renal de acordo com o sexo, Nordeste do Brasil (2008–2024).

**Tabela 2:** Distribuição dos diagnósticos histopatológicos em um registro de biópsias renais do Nordeste brasileiro.

Diagnóstico histopatológico	n	%	Mediana de idade (IIQ)	Sexo n/%	Etnia n/%
Nefrite Lúpica	876	32,9	29 (21–39)	F: 725/82,8 M: 151/17,2	Não branco: 567/64,7 Branco: 177/20,2
Glomeruloesclerose Segmentar Focal	455	17,1	25 (14–41,5)	F: 195/42,9 M: 260/57,1	Não branco: 305/67 Branco: 91/20
Nefropatia Membranosa	252	9,5	44 (31–55)	F: 102/40,5 M: 150/59,5	Não branco: 167/66,3 Branco: 50/19,8
Nefropatia por IgA	115	4,3	35 (26,5–44)	F: 51/44,3 M: 64/55,7	Não branco: 63/54,8 Branco: 35/30,4
Glomerulopatia Colapsante	106	4,0	25 (16–37)	F: 49/46,2 M: 57/53,8	Não branco: 66/62,3 Branco: 13/12,3
Doença de Lesões Mínimas	91	3,4	15 (8–27,75)	F: 35/38,5 M: 56/61,5	Não branco: 57/62,6 Branco: 25/27,5
Nefropatia Diabética	90	3,4	54 (45–62)	F: 39/43,3 M: 51/56,7	Não branco: 60/66,7 Branco: 17/18,9
Doença de Lesões Mínimas/Glomeruloesclerose Segmentar Focal	67	2,5	21 (9,5–37)	F: 28/41,8 M: 39/58,2	Não branco: 37/55,2 Branco: 25/37,3
Glomerulonefrite Difusa Aguda	56	2,1	26 (15–44)	F: 25/44,6 M: 31/55,4	Não branco: 36/64,3 Branco: 12/21,4
Vasculite Sistêmica	55	2,1	43 (24–56)	F: 32/58,2 M: 23/41,8	Não branco: 34/61,8 Branco: 10/18,2
Necrose Tubular Aguda	50	1,9	47 (22–60)	F: 33/66 M: 17/34	Não branco: 30/60 Branco: 9/18
Glomerulonefrite Crescêntica	35	1,3	42 (23,5–50)	F: 19/54,3 M: 16/45,7	Não branco: 26/83,9 Branco: 5/16,1
Gamopatia Monoclonal	35	1,3	56 (45–63,5)	F: 21/60 M: 14/40	Não branco: 20/57,1 Branco: 10/28,6
Microangiopatia Trombótica	34	1,3	30 (16,75–36,75)	F: 20/58,8 M: 14/41,2	Não branco: 28/82,4 Branco: 5/14,7
Glomerulonefrite Esclerosante Crônica	25	0,9	41 (24–52)	F: 12/48 M: 13/52	Não branco: 19/76 Branco: 6/24
Glomerulonefrite Membranoproliferativa	24	0,9	44,5 (20,25–58,5)	F: 10/41,7 M: 14/58,3	Não branco: 14/58,3 Branco: 9/37,5
Nefroesclerose Benigna	20	0,8	44 (34,75–57)	F: 8/40 M: 12/60	Não branco: 13/65 Branco: 4/20
Nefropatia Crônica	18	0,7	33,5 (16,25–44)	F: 11/61,1 M: 7/38,9	Não branco: 14/77,8 Branco: 4/22,2
Aterosclerose	10	0,4	32,5 (21–38,75)	F: 5/50 M: 5/50	Não branco: 6/60 Branco: 4/40
Esclerose Mesangial Difusa	7	0,3	5 (1,5–11)	F: 1/14,3 M: 6/85,7	Não branco: 4/57,1 Branco: 3/42,9
Outros	11	0,4	39 (24,25–47,75)	F: 7/63,6 M: 4/36,4	Não branco: 4/36,4 Branco: 3/27,3

Abreviaturas – IIQ: intervalo interquartil; M: masculino, F: feminino

A distribuição de acordo com a classe de nefrite lúpica apresentou maior frequência da classe IV (51,5%), seguida pela classe V (19,7%) (Figura 3).

**Figura 3:** Frequência das classes de nefrite lúpica em amostras de biópsia renal, Nordeste do Brasil (2008–2024).

A Tabela 3 descreve as principais características dos dois diagnósticos mais prevalentes no estudo: GESF e nefrite lúpica. Enquanto a GESF apresenta distribuição semelhante em quase todas as faixas etárias e entre os sexos, a nefrite lúpica concentra 89% dos casos na faixa etária de 15–59 anos, sendo 83% mulheres. A GESF manifestou-se principalmente como síndrome nefrótica (72%), enquanto a nefrite lúpica apresentou manifestações clínicas diversas, incluindo casos agudos, como glomerulonefrite rapidamente progressiva em 6,2% dos casos.

**Tabela 3:** Características demográficas e clínicas dos dois diagnósticos mais prevalentes em um registro de biópsias renais da região Nordeste do Brasil (2008–2024): glomeruloesclerose segmentar e focal e nefrite lúpica.

Características	Glomeruloesclerose segmentar e focal (n = 455)	Nefrite lúpica (n = 876)
**Faixa Etária (anos)**		
≤ 14	124 (27%)	74 (8,5%)
15–29	131 (29%)	367 (42%)
30–59	164 (36%)	413 (47%)
≥ 60	32 (7,1%)	16 (1,8%)
**Sexo**		
Feminino	195 (43%)	725 (83%)
Masculino	260 (57%)	151 (17%)
**Cor da Pele**		
Não branco	305 (77%)	567 (76%)
Branco	91 (23%)	177 (24%)
**Síndrome**		
Lesão Renal Aguda	4 (1,0%)	19 (2,5%)
Doença Renal Crônica	36 (9,2%)	54 (7,1%)
Anormalidades Urinárias Assintomáticas	29 (7,4%)	142 (19%)
Síndrome Nefrítica	10 (2,5%)	121 (16%)
Síndrome Nefrótica	283 (72%)	219 (29%)
Síndrome Nefrítica/ Nefrótica	24 (6,1%)	153 (20%)
Glomerulopatia Rapidamente Progressiva	6 (1,5%)	47 (6,2%)
Outros	1 (0,3%)	4 (0,5%)

## DISCUSSÃO

Apenas alguns estudos abordaram as características epidemiológicas de pacientes com doenças glomerulares na região Nordeste do Brasil^5,8^. As características sociodemográficas específicas dessa região, por sua vez, tornam importante o desenvolvimento de registros de biópsias renais nessa área como referência para comparação com outras regiões do país e até mesmo com populações em outras partes do mundo.

Devido ao alto custo e ao significativo impacto social das doenças renais, países como o Japão^14^ e a Itália^15^ mantêm regularmente registros nacionais de doenças glomerulares, algo difícil de aplicar no Brasil, por se tratar de um país continental com considerável variabilidade sociodemográfica entre as regiões^16,17^. Utilizando uma abordagem populacional de grande porte, caracterizamos pacientes submetidos à biópsia renal em hospitais públicos do Nordeste do Brasil. Dos 2.911 pacientes inicialmente selecionados, o diagnóstico não foi possível em 8,58%, uma taxa inferior às taxas relatadas em outros estudos, que variaram de 12% a 16%^4,5,10^.

A A distribuição segundo o sexo foi semelhante à observada foi semelhante à observada no Registro Brasileiro de Biópsias Renais de 2010^7^. Os pacientes afetados eram geralmente adultos e jovens adultos, em consonância com o perfil demográfico relatado em outros estudos^5,7,9,10^, o que pode ser explicado pela prevalência das principais doenças glomerulares nessa faixa etária, como nefrite lúpica e glomeruloesclerose segmentar e _focal_18,19_._

A glomerulopatia colapsante (GC) afeta desproporcionalmente indivíduos de ascendência africana, um fenômeno amplamente atribuído à presença de variantes de risco do gene APOL1, que aumentam significativamente a suscetibilidade ao desenvolvimento dessa condição^20^. Além do componente genético, a GC pode ser desencadeada por diversos fatores ambientais e clínicos, incluindo infecções virais (HIV, parvovírus B19 e arbovírus), uso de certos medicamentos, doenças autoimunes, neoplasias, eventos isquêmicos (como microangiopatia trombótica, lesões peri-infarto e embolia de colesterol) e fatores associados ao transplante renal^21^. No presente estudo, 106 (4%) pacientes foram diagnosticados com GC. Esses achados reforçam a relevância da GC em contextos nacionais e adicionam uma estimativa da prevalência dessa doença na Região Nordeste do Brasil^22^.

A síndrome nefrótica foi predominante entre os idosos, sendo a nefropatia membranosa o diagnóstico mais comum nesse grupo. Estudos publicados no Brasil^23^ e no Reino Unido^24^ relataram achados semelhantes, contrastando com dados publicados nos Estados Unidos^25^ e na Espanha^26^, nos quais a lesão renal aguda foi a mais comum, e o diagnóstico mais prevalente foi glomerulopatia crescêntica e pauci-imune. No entanto, as principais indicações para biópsia renal nessa faixa etária são hematúria, proteinúria assintomática, síndrome nefrótica, glomerulonefrite rapidamente progressiva, lesão renal aguda ou doença renal crônica idiopática^24,27^. A associação entre nefropatia membranosa e malignidade na população idosa é bem conhecida^28^.

No Nordeste do Brasil^5,7,8,9,10^ e no mundo^29,30,31^, a principal indicação para biópsia renal é a síndrome nefrótica^32^. A literatura sobre síndrome nefrótica indica que 60% a 70% dos casos têm uma etiologia primária, sendo que a GESF e a nefropatia membranosa são os diagnósticos mais frequentes em pacientes adultos e idosos^33^, enquanto a doença de lesões mínimas e a GESF predominam em crianças e adolescentes, representando 80% dos casos^34^. A NL foi a patologia mais prevalente entre todas as outras indicações para biópsia renal.

A glomerulopatia primária foi mais comum do que a glomerulopatia secundária, sendo a GESF mais prevalente entre os casos de glomerulopatia primária e a NL entre os casos de glomerulopatia secundária. Esses dados são semelhantes aos relatados em diversos estudos nacionais^4,5,7,8^ e na América Latina^35,36,37^. A GESF é o achado mais frequente em países americanos, incluindo os Estados Unidos^18^ e o Brasil^7,10^.

Na Ásia e na Europa, a nefropatia por imunoglobulina (Ig) A é a glomerulopatia primária mais comum^36,37^. Embora tenha apresentado um aumento mais acentuado na prevalência nos últimos 15 anos no Brasil^7^, a nefropatia por IgA foi identificada em < 5% da amostra. Essa prevalência é inferior à estimativa nacional^7^ e à de outro estudo realizado no estado de São Paulo (Brasil)^4^, que foi de aproximadamente 10%. Em comparação com estudos internacionais, a incidência é ainda maior, variando de 20% a 45%^29,35,36,38^. Fatores relacionados à colonização e à etnia na região Nordeste, onde há maior prevalência de afrodescendentes—população na qual a prevalência de nefropatia por IgA é menor^39^—, além da falta de triagem associada ao atraso no encaminhamento ao nefrologista e aos critérios de indicação para biópsia aplicados nos serviços hospitalares, podem explicar os resultados relatados^37^. A alta prevalência de pacientes afetados por NL pode estar relacionada às características étnicas da amostra, com a maioria dos pacientes sendo de etnia não branca^40^.

Os tipos histológicos mais prevalentes de NL foram as classes IV e V, o que constituiu um achado comum relatado em uma revisão publicada em 2018^41^. A predominância dessas classes pode ser explicada pelos critérios de seleção, segundo os quais a biópsia renal é geralmente realizada em pacientes com envolvimento renal mais grave, o que é típico dessas classes.

Observou-se uma baixa incidência de glomerulonefrite pós-infecciosa, embora seja uma causa importante de glomerulopatia em países subdesenvolvidos, considerando que o Nordeste é a região mais pobre do país, onde as condições sanitárias podem ser precárias. Isso pode ser explicado pelo seu curso frequentemente benigno, com manifestações renais tardias no curso do processo infeccioso e, muitas vezes, sem indicação para biópsia renal^42^.

A baixa prevalência de doenças genéticas, como a síndrome de Alport, pode ser explicada pela falta de padronização de métodos específicos, como testes moleculares e ME^43,44^.

A hipertensão arterial primária não constitui indicação para biópsia renal, e a detecção de nefrosclerose hipertensiva nos resultados de biópsia renal é incomum, ocorrendo em 1% a 3,5% dos pacientes^7,14,36^, semelhante aos resultados do nosso estudo. A hipertensão arterial é a segunda principal causa de DRC na Europa e a principal causa no Brasil^45^.

A glomerulonefrite crescente ocorre principalmente na glomerulopatia secundária (90%), sendo a nefrite lúpica e a doença pauci-imune as etiologias mais comuns, conforme relatado na literatura^46,47^.

As limitações do presente estudo incluem a falta de integração dos sistemas utilizados pelos centros participantes, o que dificultou o acesso às informações prognósticas dos pacientes a partir de um único prontuário eletrônico, bem como a ausência de um protocolo padronizado para a realização de microscopia em todas as amostras de biópsia renal. Também foi observada a ausência de alguns dados demográficos, o que destaca a necessidade de registros integrados entre os centros.

Este foi o primeiro estudo multicêntrico desse tipo na região Nordeste do Brasil a descrever as características clínicas e epidemiológicas de pacientes com glomerulopatias. Nossos resultados lançam as bases para o desenvolvimento de um futuro registro de glomerulopatias para a região. O desenvolvimento futuro de uma ferramenta computacional para otimizar as solicitações de biópsia renal poderá, futuramente, auxiliar na padronização das informações relevantes para estudos populacionais.

## CONCLUSÃO

A presença de glomerulopatia foi semelhante entre homens e mulheres, sendo a síndrome nefrótica a indicação mais comum para biópsia renal. Em contraste com a maioria dos estudos realizados no Brasil, a nefrite lúpica (NL) foi a glomerulopatia mais prevalente, seguida pela glomeruloesclerose segmentar e focal (GESF). Os achados deste estudo diferem dos registros do Sudeste e do Sul do Brasil em relacção à baixa prevalência de nefropatia por IgA. O perfil das glomerulopatias reflete as indicações para biópsia renal e a heterogeneidade da população brasileira, com diversas características étnicas e socioeconômicas. Os resultados deste estudo podem contribuir para uma melhor compreensão da epidemiologia das glomerulopatias no Brasil.

## Data Availability

Os conjuntos de dados gerados e/ou analisados durante o presente estudo estão disponíveis mediante solicitação razoável ao autor correspondente.
